# Multiplex cytokine levels of aqueous humor in acute primary angle-closure patients: fellow eye comparison

**DOI:** 10.1186/s12886-016-0182-8

**Published:** 2016-01-09

**Authors:** Shaolin Du, Wenbin Huang, Xiulan Zhang, Jiawei Wang, Wei Wang, Dennis S. C. Lam

**Affiliations:** Zhongshan Ophthalmic Center, State Key Laboratory of Ophthalmology, Sun Yat-Sen University, Guangzhou, China; Tungwah Hospital of Sun Yat-Sen University, Dongguan, China

**Keywords:** Cytokines, Angle-closure, Aqueous humor

## Abstract

**Background:**

The existing literature contains no information regarding inflammatory cytokine expression in unilateral acute primary angle-closure (APAC) affected eyes and fellow eyes with primary angle closure suspect (PACS). To measure levels of various inflammatory cytokines in the aqueous humor (AH) of APAC affected eyes and fellow eyes with a diagnosis of PACS (18 unilateral APAC eyes and 18 fellow eyes with PACS), and determine the underlying correlation between them.

**Methods:**

The total levels of 12 cytokines including granulocyte colony-stimulating factor (G-CSF), interleukin (IL)-6, IL-8, monocyte chemotactic protein (MCP)-1, MCP-3, macrophage-derived chemokine (MDC), macrophage inflammatory protein (MIP)-1β, and vascular endothelial growth factor (VEGF) etc. were assessed using the multiplex bead immunoassay technique. The level of cytokines in different groups was analyzed by a 2-related-samples nonparametric test. Data on patient demographics, preoperative intraocular pressure (IOP), number of glaucoma medications, as well as several ocular biological parameters were also collected for correlation analysis.

**Results:**

The APAC patients had significantly higher levels of G-CSF, IL-6, IL-8, MCP-1, MCP-3, MDC, MIP-1β, and VEGF in the AH samples from unilateral APAC affected eyes than in fellow eyes with PACS (all *P* < 0.05). The cytokines showed positive correlations between each other (*P* < 0.0071).

**Conclusions:**

Cytokine networks in the AH may have critical roles in the progression of APAC. Thus, different cytokine expression in both eyes of the same patient may help us to understand the different pathology in APAC and PACS.

## Background

Acute primary angle-closure (APAC) presents with typical symptoms and clinical signs such as sudden onset of ocular discomfort or pain, subjective blurring of vision, and suddenly excessive increases in intraocular pressure (IOP). In APAC, the position of the iris causes the normally open anterior chamber angle to close. Aqueous humor (AH) that should normally drain out of the anterior chamber is then trapped inside the eye, increasing the IOP [1].

The AH plays an important role in the pathogenesis of glaucoma [2]. Furthermore, AH has the task of protecting and supplying nutrients and antioxidants to the cornea, lens, and trabecular meshwork (TM) [3, 4]. A number of cytokines, including transforming growth factor (TGF-β2), interleukin (IL-6, and IL-8), have been detected in the AH [5, 6]. Several studies that measured cytokine concentrations in AH samples have revealed significantly high levels in primary open angle glaucoma, exfoliation glaucoma [7], and primary angle closure glaucoma [8].

By 2020, it is estimated almost 80 million persons will have glaucoma worldwide. Particularly in Asian countries, angle closure glaucoma accounts for no less than half of the blindness arising from glaucoma. Therefore, more attention needs to be paid to APAC in China. In our previous report, compared with the cataract group, APAC showed clear and significantly elevated concentrations of IL-6, IL-8, granulocyte colony-stimulating factor (G-CSF), monocyte chemotactic protein (MCP)-1, MCP-3, and vascular endothelial growth factor (VEGF) [9]. However, the existing literature contains no information regarding inflammatory cytokine expression in unilateral APAC affected eyes and fellow eyes with primary angle-closure suspect (PACS).

In this study, we want to exlore whether some different inflammation-related cytokines expressed in APAC compared with fellow eyes with PACS and whether there are novel findings different from those previously published by Huang et al. [9]. Huang’s previous report have measured levels of various inflammation-related cytokines in the aqueous humor of patients with acute primary angle-closure (APAC) and senile cataract. The present study want to explore different inflammatory cytokine expression in unilateral acute primary angle-closure (APAC) affected eyes and fellow eyes with primary angle closure suspect (PACS). The design of fellow eye comparison may help us to find novel things. Our finding may expand the understanding of asymmetric glaucoma pathophysiology.

## Methods

### Subjects and enrollment criteria

Participants in this study were selected as a convenience sample of patients at the Glaucoma Department, Zhongshan Ophthalmic Center. Both eyes of each subject were included. All enrolled glaucoma patients fulfilled the following criteria: Subjects were > 18 years old. APAC was defined according to the following criteria: [10] ➀ Presence of at least two of the following symptoms: ocular or periocular pain, nausea and/or vomiting, and an antecedent history of intermittent blurring of vision with halos; ➁ IOP of at least 22 mm Hg (as measured by Goldmann applanation tonometry); ➂ presence of at least three of the following signs of conjunctival injection: corneal epithelial edema, mid-dilated unreactive pupil, and a shallow anterior chamber; and ➃ presence of an occluded angle in the affected eye, verified by gonioscopy. Ultrasound biomicroscopy (UBM) examination confirmed that a narrow-angle pupillary block component existed in all eyes. PACS fulfilled the following criteria: ➀narrow angles (defined as eyes in which at least 180°of the posterior pigmented trabecular meshwork was not visible on gonioscopy in the primary position of gaze without indentation); ➁ IOP less than or equal to 21 mmHg ➂ healthy optic disc; ➃ without primary angle closure [11].

The AH collection was performed under sterile conditions via an anterior chamber paracentesis or peripheral iridectomy, or before commencement of surgery for all trabeculectomy-required patients, and their fellow PACS eyes were scheduled for prophylactic surgical peripheral iridectomy according to agreements of the patients and the requirements of the Chinese medical association. These management recommendations are decided according to the American Academy of Ophthalmology Preferred Practice Pattern guidelines on primary angle closure (http://www.aao.org) and individual situations.

The antiglaucomatous medication treatment for APAC was standardized as follows: topical pilocarpine 1 % four times daily; topical beta-blocker (timolol 0.5 %) twice daily and/or brinzolamide (Azopt; Alcon Laboratories, Elkridge, MD), and/or topical alpha-2 agonists (Alphagan; Allergan, Inc., Irvine, CA); topical steroids; oral acetazolamide 250 mg three times daily and intravenous mannitol 20 % at 1 to 2 g/kg 4 h after the initiation of the treatment in cases where the IOP was not reduced by 20 % from the initial IOP, excluding contraindicated by systemic disease (e.g., congestive heart failure).

Patients who met any of the following criteria were excluded: a secondary acute attack because of lens subluxation, uveitis, iris neovascularization, trauma, tumor, or any obvious cataract leading to an intumescent lens; a known systemic inflammatory, autoimmune, or immunosuppressive disease; a pre-existing ocular disease (retinal vein occlusion, retinal artery occlusion, diabetic retinopathy, age-related macular degeneration); or a history of previous ocular surgery.

All eyes of the subjects underwent a thorough ophthalmic evaluation, including slit-lamp biomicroscopy, IOP measurement (Goldmann applanation tonometry), gonioscopy, fundus examination, UBM, and B-scanning. IOP was measured preoperatively, mainly in the afternoon on the day before the AH sampling for all studied eyes. We believe that this minimized the influences of diurnal variation of IOP and closely reflected the IOP at the time of AH sampling.

### Aqueous humor collection

The AH samples (50–100 μL) were collected by a procedure described in our previous study [12]. Two AH samples of APAC eyes were collected during an anterior chamber paracentesis procedure, and 16 samples were collected at the beginning of peripheral iridectomy or trabeculectomy. The AH samples of PACS eyes were collected at the beginning of peripheral iridectomy. All samples were obtained prior to any conjunctival or intraocular manipulation to avoid breakdown of the blood–aqueous barrier associated with surgical trauma. All samples were immediately frozen and stored at −80 °C until the analyses were performed.

### Cytokine analysis

Cytokine concentrations were analyzed using a multiplex bead immunoassay system (Milliplex Human Cytokine® kit; Millipore Corp., Billerica, MA). The assays were performed according to the manufacturer’s instructions and analyzed using the Bio-Plex suspension array system (Bio-plex200, Bio-Rad, Hercules, CA). Cytokines related to the inflammatory process [G-CSF, IL-6, IL-8, MCP-1, MCP-3, macrophage-derived chemokine (MDC), MIP-1β, VEGF, GM-CSF (granulocyte-macrophage, colony-stimulating factor), IFN-γ (interferon-gamma), IL-1βand TNF-β (tumor necrosis factor-beta)] were analyzed in a 25 μl volume of AH sample for each reaction. Based on the information provided by the manufacturer, the multiplex assay kit can quantitatively measure multiple cytokines from as little as 25 μL of body fluids. The detection limit for any analyte was 1 pg⁄ml, with a dynamic range up to 10000 pg⁄ml (according to the manufacturer).

### Statistical analysis

The data were processed and analyzed statistically using SPSS (Version 13.0; SPSS, Chicago, IL). The level of cytokines in different groups was analyzed by a 2-related-samples nonparametric test. Statistical significance was accepted at *P* < 0.05. Correlations among cytokines and correlations between cytokine concentrations and subjects’ demographic data (including age, IOP and anterior chamber depth (ACD), lens thickness (LT) and vitreous chamber depth (VCD) in the macular region) were calculated using Spearman’s correlation test. For the correction of multi-group comparisons, *P* values of 0.0071 for Spearman’s correlation test were considered statistically significant, with significance levels of 0.05 based on Bonferroni’s methods.

### Ethics

This study was approved by the Ethical Review Committee of Zhongshan Ophthalmic Center and adhered to the provisions of the Declaration of Helsinki for research involving human subjects. All subjects participating in this study were given a detailed explanation about the study and signed an informed consent form. Participants were recruited prospectively and consecutively for this study, between January 2013 and May 2014. All subjects were from a Chinese Han population.

## Results

### Patients’ demographic data

Eighteen APAC patients (18 unilateral APAC affected eyes and 18 fellow eyes with PACS) who fulfilled the inclusion criteria were included in the study.

The demographic data of the subjects, including age, sex, number, and details of glaucoma medication use, are summarized in Table 1. Biological parameters of APAC eyes and PACS eyes are summarized in Table 2. The mean age of the APAC patients was 63.80 ± 6.76 (mean ± standard deviation) years. Data for duration of experienced attack, number of glaucoma medications, IOP at the day of surgery, Axial Length (AL), ACD, LT and VCD are expressed as the median [IQ]. IOP on the day of surgery was higher in the APAC eyes than in PACS eyes as determined by a 2-related-samples nonparametric test (*P* = 0.008).

**Table 1:** Demographic and baseline characteristics of patients

Characteristics	Subjects of study
No. of patients (No. of eyes)	18 (36)
Age, y (mean [SD])	63.80 (6.76)
Sex (male/female)	5/13
Laterality of affected eye, right/left	7/11
Duration of experienced attack, d (median [IQ])	5.50 (8.25)
No. of glaucoma medications in APAC eyes, n (median [IQ])	1.00 (1.00)

*SD* standard deviation, *IQ* inter-quartile

**Table 2:** Characteristics of Acute Primary Angle-Closure Eyes and Primary Angle-Closure Suspect Eyes

Characteristics	Affected eyes, APAC	Fellow eyes, PACS	*P* value
IOP at the day of surgery, mmHg	19.5 (20.68)	13.5 (6.0)	0.008*
Axial Length (AL), mm	22.42 (1.50)	22.25 (1.26)	0.138
Anterior Chamber Depth (ACD), mm	2.24 (0.21)	2.22 (0.28)	0.236
Lens Thickness (LT), mm	5.17 (0.61)	5.29 (0.67)	0.244
Vitreous Chamber Depth (VCD), mm	14.96 (1.35)	14.91 (1.32)	0.206

Data are expressed as the median (IQ). *P*-values were obtained from 2-related-samples nonparametric test. * *P*-value < 0.05 was considered to be significant

### The comparison of cytokines between APAC eyes and PACS eyes

**Fig. 1:**
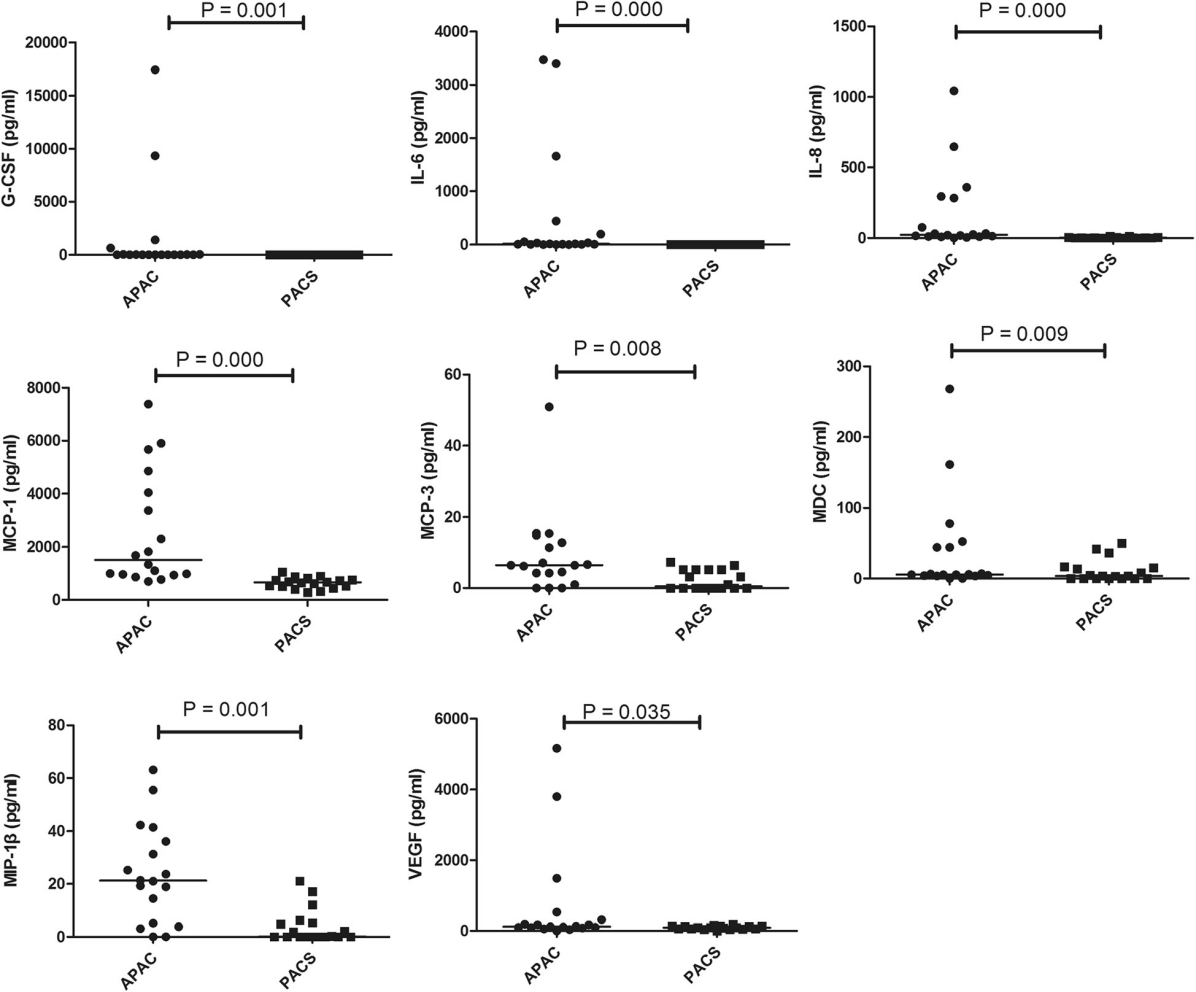
Differences of cytokines in APAC with PACS. Scatterplots showing distribution levels of G-CSF, IL-6, IL-8, MCP-1, MCP-3, MDC, MIP-1β, and VEGF in aqueous humor from unilateral acute primary angle-closure (APAC) affected eyes and fellow eyes with primary angle-closure suspect (PACS). Two-related-samples nonparametric test was performed between groups, and a significant difference was accepted at *P* < 0.05. The solid lines indicate median concentrations. In the APAC patients, the levels of G-CSF (*P* = 0.001), IL-6 (*P* = 0.000), IL-8 (*P* = 0.000), MCP-1 (*P* = 0.000), MCP-3 (*P* = 0.008), MDC (*P* = 0.009), MIP-1β (*P* = 0.001), and VEGF (*P* = 0.035) in AH samples from unilateral APAC affected eyes were significantly higher than those from fellow eyes with PACS

GM-CSF, IFN-γ, IL-1β, and TNF-β were detected in less than 50 % of samples in both control and glaucoma groups and therefore were not included in further analysis. The concentrations of the remaining eight cytokines analyzed are shown in Table 3. All measurements were performed successfully. In the APAC patients, the levels of G-CSF (*P* = 0.001), IL-6 (*P* = 0.000), IL-8 (*P* = 0.000), MCP-1 (*P* = 0.000), MCP-3 (*P* = 0.008), MDC (*P* = 0.009), MIP-1β (*P* = 0.001), and VEGF (*P* = 0.035) in AH samples from unilateral APAC affected eyes were significantly higher than those from fellow eyes with PACS (Fig. 1).

**Table 3:** Level of cytokines in aqueous humor from APAC eyes and fellow eyes with PACS

	Affected eyes, APAC (pg/ml)	Fellow eyes, PACS (pg/ml)	*P* value
G-CSF	1608.42 (13.71; 0.00, 17420.00)	1.11 (0.00; 0.00, 13.58)	0.001*
IL-6	520.24 (14.62; 0.00, 3470.00)	0.35 (0.00; 0.00, 1.82)	0.000*
IL-8	160.37 (23.28; 0.00, 1043.00)	2.96 (1.09; 0.00, 13.64)	0.000*
MCP-1	2537.39 (1507.50; 696.75, 7384.00)	641.71 (661.50; 281.49, 1051.00)	0.000*
MCP-3	9.30 (6.45; 0.00, 50.90)	2.34 (0.49; 0.00, 7.35)	0.008*
MDC	38.80 (5.60; 0.00, 268.00)	10.98 (3.61; 0.00, 50.00)	0.009*
MIP-1β	23.67 (21.21; 0.00, 63.10)	3.95 (0.16; 0.00, 21.07)	0.001*
VEGF	701.60 (120.43; 0.00, 5162.00)	93.87 (91.01; 0.00, 182.00)	0.035*

*APAC* acute primary angle-closure; *PACS* primary angle-closure suspect. Granulocyte colony-stimulating factor (G-CSF), interleukin (IL)-6, IL-8, monocyte chemotactic protein (MCP)-1, MCP-3, macrophage-derived chemokine (MDC), macrophage inflammatory protein (MIP)-1β, and vascular endothelial growth factor (VEGF). Data are expressed as the mean (median; range). *P*-values were obtained from 2-related-samples nonparametric test.* *P*-value <0.05 was considered to be significant

### The correlations among different cytokine concentrations

The correlations among the different cytokine concentrations are shown in Table 4. Among seven cytokines (G-CSF, IL-6, IL-8, MCP-1, MCP-3, MDC, and macrophage inflammatory protein (MIP-1β) positive correlations were found between each other (*P* < 0.0071). Negative correlations were found between VEGF and G-CSF (*ρ* = 0.307; *P* = 0.068), VEGF and MCP-3 (ρ = 0.434; *P* = 0.008), and VEGF and MDC (*ρ* = 0.250; *P* = 0.141).

**Table 4:** Correlations among cytokines

ρ/*P* value	G-CSF	IL-6	IL-8	MCP-1	MCP-3	MDC	MIP-1β	VEGF
G-CSF	—	0.829	0.726	0.696	0.785	0.547	0.608	0.307
IL-6	0.000*	—	0.811	0.886	0.703	0.549	0.647	0.480
IL-8	0.000*	0.000*	—	0.785	0.715	0.647	0.841	0.512
MCP-1	0.000*	0.000*	0.000*	—	0.649	0.461	0.631	0.515
MCP-3	0.000*	0.000*	0.000*	0.000*	—	0.500	0.607	0.434
MDC	0.001*	0.001*	0.000*	0.005*	0.002*	—	0.461	0.250
MIP-1β	0.000*	0.000*	0.000*	0.000*	0.000*	0.005*	—	0.472
VEGF	0.068	0.003*	0.001*	0.001*	0.008	0.141	0.004*	—

Granulocyte colony-stimulating factor (G-CSF), interleukin (IL)-6, IL-8, monocyte chemotactic protein (MCP)-1, MCP-3, macrophage-derived chemokine (MDC), macrophage inflammatory protein (MIP)-1β, and vascular endothelial growth factor (VEGF)

Correlation coefficient (ρ) and *P* values are calculated by Spearman’s correlation

*Significance level at 5 % (*P* < 0.0071) by Bonferroni correction for multiple comparisons

### The correlations between each cytokine and age, IOP, the number of glaucoma medications, and some ocular biological parameters in APAC patients

The correlations between each cytokine and age, IOP, number of Glaucoma Medications, AL, ACD, LT, and VCD in APAC patients are shown in Table 5. Positive correlations existed between IOP and G-CSF (*ρ* = 0.506; *P* = 0.002). The number of glaucoma medications was positively correlated with G-CSF (*ρ* = 0.464; *P* = 0.004), IL-6 (*ρ* = 0.443; *P* = 0.007), and IL-8 (*ρ* = 0.439; *P* = 0.007).

**Table 5:** Correlations between Cytokines and relevant factors in APAC patients

	Age		IOP		Number of Glaucoma Medications		AL		ACD		LT		VCD	
	ρ	*P* value	ρ	*P* value	ρ	*P* value	ρ	*P* value	ρ	*P* value	ρ	*P* value	ρ	*P* value
G-CSF	−0.232	0.173	0.506	0.002*	0.464	0.004*	−0.060	0.729	−0.074	0.670	0.121	0.482	−0.097	0.572
IL-6	−0.115	0.503	0.424	0.010	0.443	0.007*	−0.089	0.605	0.031	0.860	0.040	0.815	−0.132	0.444
IL-8	−0.143	0.406	0.375	0.024	0.439	0.007*	−0.198	0.246	0.056	0.743	0.004	0.980	−0.201	0.239
MCP-1	−0.097	0.573	0.291	0.085	0.285	0.092	−0.110	0.523	0.040	0.817	−0.002	0.989	−0.140	0.414
MCP-3	−0.127	0.460	0.420	0.011	0.347	0.038	−0.040	0.816	0.005	0.977	0.177	0.300	−0.108	0.531
MDC	−0.261	0.125	0.201	0.240	0.131	0.445	−0.411	0.013	0.193	0.259	−0.182	0.288	−0.364	0.029
MIP-1β	−0.166	0.335	0.387	0.020	0.395	0.017	−0.130	0.450	0.003	0.985	0.029	0.865	−0.126	0.463
VEGF	0.013	0.941	0.318	0.058	0.198	0.247	−0.051	0.769	−0.091	0.597	0.300	0.075	−0.185	0.280

Relevant factors: Age, intraocular pressure (IOP), Number of Glaucoma Medications, Axial Length (AL), Anterior Chamber Depth (ACD), Lens Thickness (LT), Vitreous Chamber Depth (VCD) and Choroidal Thickness in the Macular Region (CT)

Granulocyte colony-stimulating factor (G-CSF), interleukin (IL)-6, IL-8, monocyte chemotactic protein (MCP)-1, MCP-3, macrophage-derived chemokine (MDC), macrophage inflammatory protein (MIP)-1β, and vascular endothelial growth factor (VEGF)

Correlation coefficient (ρ) and *P* values are calculated by Spearman’s correlation

*Significance level at 5 % (*P* < 0.0071) by Bonferroni correction for multiple comparisons

## Discussion

The bead immunoassay revealed that the levels of G-CSF, IL-6, IL-8, MCP-1, MCP-3, MDC, MIP-1β, and VEGF were significantly higher in AH samples from unilateral APAC affected eyes than from fellow eyes with PACS. Clear elevations of cytokines related to an immune reaction or inflammation existed in AH samples from APAC eyes. This is one of the earliest studies to report successful detection of multiple cytokines with the method of the multiplex bead immunoassay in AH samples from APAC and PACS eyes. A previous study showed that normal TME cells constitutively secreted chemotactic cytokines like IL-8 and MCP-1. Secretion of these chemokines was augmented by treatment with the pro-inflammatory cytokines or other stimulations [13].

IL-8, also called chemokine (C-X-C motif) ligand-8 (CXCL8), is a member of the CXC family of chemokines and is known to have both immune and vascular functions [14, 15]. Multiple studies have demonstrated an elevation of IL-8 under suboptimal oxygenation conditions, which suggests a possible mechanism for the increased IL-8 found in our study of APAC patients [5, 16]. The concentration of the inflammatory cytokine IL-8 was significantly elevated in the AH of primary open angle glaucoma (POAG) patients, supporting the hypothesis that immune activation occurs during glaucoma [5, 7]. A study by Kuchtey et al. also demonstrated a significantly higher IL-8 concentration in the more severely affected eyes of the patients with asymmetric glaucoma [5]. The present study showed that IL-8 was higher in the APAC eyes than the fellow eyes with PACS. In an acute attack of glaucoma immunological activation probably has nothing to do. It is evident that if the iris touches the trabecular meshwork occurs inflammation, but then it is possible that this will stimulate the production of this cytokine because the outflow is hampered. In fact, IL-8 modulates the permeability of the Schlemm’s canal endothelial cells reported by Alvarado et al. [17]. Therefore, it is more plausible that the IL-8 presence in APAC eyes is due to the attempt by the trabecular meshwork to increase the outflow is hampered [18].

Furthermore, monocytes, presumably under the influence of chemotactic signals, circulate through the trabecular meshwork in the normal state and also that cytokines regulate the permeability of Schlemm’s canal endothelial cells [19]. Therefore, the increase of MCP-1 should be viewed in the same way or to attempt to do work better than the outflow of the trabecular meshwork. Also the granulocyte colony-stimulating factor is produced by the TM endothelial cells and probably works as a reminder for the stem cells of the TM, because in an acute attack of glaucoma occurs severe suffering of the TM endothelial cells.

VEGF was another important cytokine investigated in this study, and its level was significantly higher in APAC eyes. Previous studies have detected VEGF protein in AH from eyes with POAG, angle closure glaucoma, and exfoliation glaucoma (EXG) [20]. Although the study by Takai et al. reported a mean difference in the level of VEGF that did not reach significance, the level was higher in the EXG group [7]. Another study reported a significant association between VEGF and the final IOP in patients with POAG [21]. The study by Kim et al. reported that the level of VEGF in the AH was significantly higher in the failure group after the Ahmed glaucoma implantation compared with the success group, implying that VEGF may play a role in determining surgical success after Ahmed valve implantation in patients with neovascular glaucoma (NVG) [22]. VEGF expression can be induced by subjecting cells to hypoxia or hypoglycemia [23]. In this present study, our findings for VEGF suggest that an anoxic environment possibly plays a key role in the pathogenesis of glaucoma and in surgical success.

The present study also indicated significant differences in the levels of IL-6, MDC, and MIP-1β between APAC eyes and PACS eyes. Some past studies found no significant correlations for IL-6 levels in AH from POAG patients [7, 24]. Interleukin-6 (IL-6) is a multi-functional cytokine that regulates immune responses, acute phase reactions, and hematopoiesis, and may play a central role in host defense mechanisms. MDC is produced by both immune and nonimmune cells, usually in response to inflammatory stimuli or tissue damage. The study by Yin et al. suggested that macrophage-derived factors stimulate optic nerve regeneration [25]. MIP-1β, which induces both chemotaxis and adhesion of T cells [26], may play a key role in an immune reaction in the APAC eyes.

The significant correlations among the cytokines (G-CSF, IL-6, IL-8, MCP-1, MCP-3, MDC and MIP-1β) suggested that the cytokine networks play important roles in inflammation and IOP elevations in APAC, although the exact mechanism of the interactions among these cytokines are unclear. Therefore, evaluation of the AH composition of various inflammatory cytokines may expand the understanding of APAC pathophysiology.

Other parameters, such as axial length of the eye, age, and gender, may also affect the measured cytokine data [27]. In the present study, the data for age, IOP, number of glaucoma medications, AL, ACD, LT and VCD were analyzed for correlations and statistically significant differences were noted between G-CSF and IOP, G-CSF and number of glaucoma medications, IL-6 and number of glaucoma medications, and IL-8 and number of glaucoma medications.

Although the exact mechanism of this interaction is unclear, the IOP levels suggested a strong correlation between G-CSF and IOP elevation. Recently, Freedman and Iserovich [28] have reported that IOP is a potential stimulus for cytokine production. Huang’s report [9] suggests that the IOP itself may be responsible for the production of cytokines. However, in this study the finding was only G-CSF was correlated with IOP. That’s different with the findings by Huang et al. who have found that IL-8, MCP-3, and VEGF also correlated with IOP besides G-CSF. Lots of reports suggest that the relationship between IOP and inflammation is complex. IOP elevation may cause disruption of the blood–aqueous barrier, thereby resulting in production of cytokines in aqueous humor [29]. The inflammatory cells and proteins can result in the formation of posterior synechiae leading to peripheral anterior synechiae and even IOP elevation [30]. To analyze the deep reasons for the differences with previous studies, the main reason maybe the high-efficiency of statistical method. It’s obvious that fellow eye comparison can test the results more effectively than other way. The present study may suggest PACS eyes G-CSF has played more important role than other cytokines in APAC eyes, and other influencing factors may also play important roles.

Previous studies have revealed that conjunctival inflammation can be developed in eyes treated with topical antiglaucoma medication [31]. Particularly, conjunctival inflammatory infiltrates may be caused by high cumulative antiglaucoma treatments [32, 33]. Similar histopathological effects of antiglaucomatous drugs may also happened in the trabecular meshwork [34]. What about the effects of topical glaucoma medications on inflammatory cytokine expression? Engel et al’s reported that preoperative IOP-lowering eye drops did not significantly alter the anterior chamber milieu in POAG [35]. The number of glaucoma medications was positively correlated with G-CSF, IL-6, and IL-8. Topical glaucoma medications may take more important effects on conjunctival inflammation than the inflammation in aqueous humor.

Lastly, in a study such as the present one, which involve the simultaneous analysis of multiple factors from each sample, some factors such as sample size and method of data analysis may affect the outcome. The Bonferroni correction was applied in the present case. This is a conservative statistical adjustment used for the verification of data observations but it may mask potential changes in exploratory studies. Some potential limitations in our study should also be mentioned. Use of topical anti-glaucoma medications may influence the aqueous immune milieu [7]. Another limit of the study is the sample size: the number of patients enrolled into our study was relatively low. Nevertheless, the results were statistically significant, so the relatively small number of patients may serve to strengthen the results and conclusions of the study.

## Conclusions

In conclusion, the present study showed that APAC is associated with changes in the AH cytokine profile. These findings may support the hypothesis that inflammation and hypoxia may play a key role in the pathogenesis of glaucoma. Thus, different cytokine expression in both eyes of the same patient may help us to understand the different pathology in APAC and PACS. However, we can not explain why PACS do not develop into APAC at the same time or some PACS eyes do not develop into APAC forever. Further studies will be necessary to resolve these issues.

## Abbreviations

APAC: Acute primary angle-closure
IOP: Intraocular pressure
AH: Aqueous humor
TM: Trabecular meshwork
TGF-β2: Transforming growth factor
IL: Interleukin
G-CSF: Granulocyte colony-stimulating factor
MCP: Monocyte chemotactic protein
VEGF: Vascular endothelial growth factor
PACS: Primary angle closure suspect
UBM: Ultrasound biomicroscopy
TNF-β: Tumor necrosis factor-beta
ACD: Anterior chamber depth
LT: Lens thickness
VCD: Vitreous chamber depth
AL: Axial length
MDC: Macrophage-derived chemokine
MIP: Macrophage inflammatory protein
CXCL: Chemokine (C-X-C motif) ligand
POAG: Primary open angle glaucoma
NVG: Neovascular glaucoma
